# Tumor-Bowel Fistula as a Rare Form of Recurrent Ovarian Cancer—Imaging and Treatment: Preliminary Report

**DOI:** 10.3390/curroncol30010040

**Published:** 2022-12-29

**Authors:** Melania Jankowska-Lombarska, Laretta Grabowska-Derlatka, Pawel Derlatka

**Affiliations:** 1 Second Department of Clinical Radiology, Medical University of Warsaw, Banacha 1a St., 02-097 Warsaw, Poland; 2 Second Department Obstetrics and Gynecology, Medical University of Warsaw, Karowa 2 St., 00-315 Warsaw, Poland

**Keywords:** recurrent ovarian cancer, tumor-bowel fistula, computed tomography (CT), magnetic resonance imaging (MRI), surgical treatment

## Abstract

Background. The aim of this pilot study was to evaluate the value of imaging techniques, including computed tomography (CT) and magnetic resonance imaging (MRI), in the diagnosis of a tumor-bowel fistula as a rare form of epithelial ovarian cancer (EOC) relapse. We also performed an initial assessment of the effectiveness of the treatment of this form of relapse. Methods. The study group consisted of eight patients with suspected platinum-sensitive recurrence in the form of a tumor/bowel fistula. All patients finished their first line of chemotherapy and subsequently showed complete remission for 6 months or more. To qualify patients for further treatment, CT and MRI were performed, which suggested the presence of a fistula between the recurrent tumor and intestine. DESKTOP study criteria were used to qualify patients for secondary cytoreduction. Second-line chemotherapy was given after secondary debulking. Results. In all patients, fistulas formed between the tumor and large bowel. On CT, the fistulas were indirectly visible. In all cases, the fistula was visible on MR images, which showed hypointensity on the T2 and T1 post-contrast sequences but did not show restricted diffusion on the diffusion-weighted imaging (DWI) sequence. Patients who were qualified for the study underwent secondary debulking with bowel resection. In all eight cases, the fistula between the tumor and surrounding organs was confirmed. During surgery, seven intestinal anastomoses and one colostomy were performed. No residual macroscopic tumor remained in seven cases (resection R0-87.5%). The progression-free survival (PFS) was 8.4–22.6 months (median 13.4). In the group with cytoreduction R0, the median PFS was 15.5 months (12–22). Conclusion. In patients with suspected EOC recurrence with clinically suspected fistula, CT scan is not sufficient. In CT, the presence of a fistula is suspected based on indirect symptoms. MRI, as a method with much greater tissue resolution, confirms the diagnosis. In addition, MRI can identify the point of the tumor/bowel junction. This is especially true with a large infiltration covering several intestinal parts. Bowel resection with simultaneous anastomosis is a good and safe solution for these patients. However, appropriate qualification for the procedure is necessary, which will allow for surgery without residual macroscopic disease (R0 surgery). Due to the small number of cases, our results cannot be generalized. We treat them as a hypothesis that can be verified in a larger study.

## 1. Introduction

Epithelial ovarian cancer (EOC) is the fifth most common cancer in women and is also the fourth leading cause of death from cancer because of the lack of discernible symptoms and effective screening tools [1,2]. Tumor prognosis depends on the use of optimal cytoreductive surgery and adjuvant platinum-based chemotherapy [3,4]. Current maintenance treatment with bevacizumab or a polyadenosine diphosphate-ribose polymerase (PARP) inhibitor has been associated with longer progression-free survival (PFS) [5,6]. The standard of care for relapsed cancer has mainly been systemic treatment. However, a few trials, especially the DESKTOP I-IIII series, have shown a significant overall survival benefit with cytoreductive surgery followed by chemotherapy in patients with recurrent platinum-sensitive ovarian cancer compared with patients who received chemotherapy alone [7,8,9].

The risk of recurrence in ovarian cancer depends on the stage of the disease at the time of diagnosis and post-operative residual tumor; however, overall, 70% of patients with advanced disease will experience recurrence [10]. Relapsing ovarian cancer can manifest as localized or widespread recurrence. Fistula formation is frequently associated with relapsed ovarian cancer [11].

A fistula is an abnormal connection between two epithelial surfaces, such as hollow organs, skin, and vessels. In the female pelvis, fistulas can develop in various locations; however, the most common sites are the enterovaginal, colonovaginal, rectovaginal, and vesicovaginal sites. In malignancy, their formation is a rare complication of radiation therapy and surgery and a rare symptom of primary or recurrent tumors, such as ovarian, cervical, and rectal cancer. Spontaneous rectovaginal fistula has also been reported in women with primary and recurrent ovarian cancer undergoing bevacizumab therapy [12]. Rarely due to a large size, a tumor can infiltrate local tissues such as the intestine, bladder, and vagina and form a fistula between the mass of the tumor and adjacent organs. Radiological imaging by magnetic resonance and computed tomography are key in the diagnosis and further treatment of pelvic fistulas [13].

Usually, the first imaging technique used for suspected ovarian cancer is transvaginal ultrasound, as it is accessible. It is also often used to monitor patients after surgeries, chemotherapy, and radiation. To confirm or reject the suspicion of ovarian cancer, other imaging techniques need to be used, such as magnetic resonance and computed tomography. Magnetic resonance is the most important technique for imaging of the female pelvis, as it provides the best possible soft tissue resolution [14]; thus, it is useful for determining local spread of the tumor and post-treatment, radiation, and surgical complications. Computed tomography is usually used as a first method to monitor progression or regression of malignant disease after surgery, radiotherapy, or chemotherapy [15].

The aim of this pilot study was to evaluate the value of imaging techniques, including computed tomography and magnetic resonance imaging, in the diagnosis of a rare form of ovarian cancer recurrence, tumor-bowel fistula. We also performed an initial assessment of the effectiveness of the treatment of this form of relapse.

## 2. Methods

A single-center study was conducted at the Medical University of Warsaw in the 2nd Department of Clinical Radiology and the 2nd Department Obstetrics and Gynecology. From 2016 to 2021, the study included 8 patients aged 37–78 years with recurrent ovarian cancer and suspected platinum-sensitive recurrence due to their clinical symptoms (e.g., bowel obstructions, rectal bleeding, and abdominal pain), transvaginal ultrasound results, and elevated CA 125 values. All patients completed their first line of chemotherapy and subsequently showed complete remission for 6 months or more. To qualify patients for further treatment, computed tomography and magnetic resonance imaging were performed, which suggested the presence of a fistula between the recurrent tumor and intestine or urinary bladder in all the patients. The clinical data of the patient population are summarized in Table 1.

### 2.1. Imaging Technique

#### 2.1.1. CT Protocol

All patients underwent computed tomography in the supine position. All patients were examined in a GE computed tomography 64-row scanner with the following scanning parameters: the axial slice thickness of the images was 1.25–2.5 mm, the electric current was 509 mA, and the electrical potential was 120 kV. Some of the parameters are shown in Table 2. Two radiologists experienced in pelvic CT evaluated images of the fistula and its type. The protocol included a precontrast phase and arterial and venous phases. Postcontrast phases were obtained after administering 70–90 mL of the intravenous low-osmolality iodinated contrast with a constant injection velocity 4 mL/s.

#### 2.1.2. MRI Protocol

All patients underwent MR imaging using a 1.5 T clinical whole-body MR system (MAGNETOM Avanto; Siemens AG, Erlangen, Germany).

The MRI protocol for the abdomen and pelvis included turbo spin-echo (TSE), T2-weighted images (T2w), fat-suppressed T2-weighted and T1-weighted images (fsT2w and fsT1w), diffusion-weighted echo planar imaging (DW-EPI), and pre- and post-contrast dynamic T1-weighted gradient echo (3D T1 GRE) sequences. Some of the MRI parameters are shown in Table 3. 

Two radiologists experienced in pelvic MRI evaluated images of the fistula and its type. In all patients, gadobutrol (Gadovist, Bayer Schering, Berlin, Germany) was administered as a bolus dose of 0.1 mmol/kg, which was immediately followed by a bolus dose of 20 mL of physiological saline (NaCl 0.9%). Interobserver agreement was assessed using the kappa coefficient.

### 2.2. Treatment Protocol

DESKTOP study criteria were used to qualify patients for secondary cytoreduction. Among the criteria, a good general condition according to the Eastern Cooperative Oncology Group (ECOG)-0, residual disease after primary surgery-0 mm, and ascites <500 mL were included [7]. Surgery treatment was performed by a team of experienced gynecologic oncologists. Considering the possibility of extensive surgery consisting in resection of the intestine, pre-operative and post-operative parenteral nutrition was used in each patient.

Second-line chemotherapy was given after secondary cytoreduction surgery according to the following regimen: two patients received liposomal doxorubicin (30 mg/m^2^) with carboplatin (AUC 5), and six patients received paclitaxel (175 mg/m^2^) with carboplatin (AUC 6) every three weeks. Two patients with BRCA1 mutation were given olaparib 2 × 300 mg (days 1–28) as maintenance treatment.

## 3. Results

### 3.1. Imaging Findings

**Figure 1:**
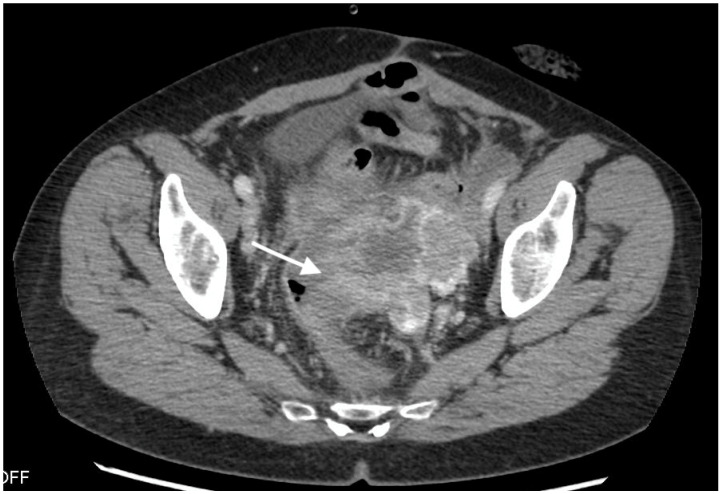
Images from 58-year-old patient with carcinosarcoma of the ovary and recurrence in the rare form of giant cell carcinoma. The fistula between tumor and rectum (arrow), CT post-contrast image in venous phase obtained in the axial plane, shows pelvic tumor and adjacent intestine, fatty tissue between the tumor and intestine vanished as indirect sign of the fistula.

**Figure 2:**
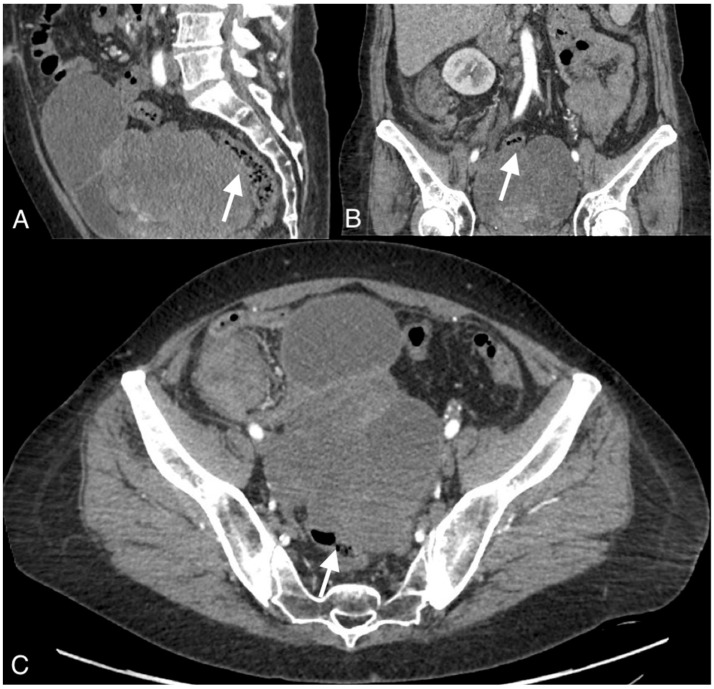
Images of a 54-year-old patient with an ovarian cancer recurrence in the form of the fistula between the tumor and rectum. CT obtained in the coronal (**A**), sagittal (**B**), and axial plane (**C**) shows large pelvic tumor with adjacent rectum.

**Figure 3:**
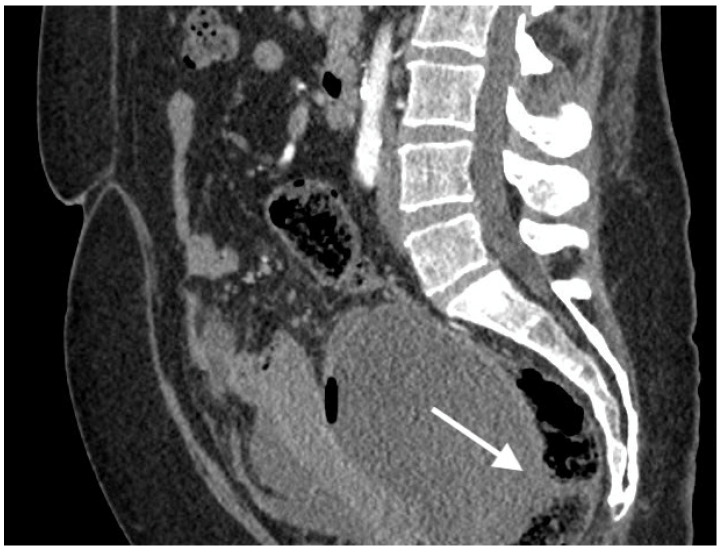
Images of a 57-year-old patient with high-grade serous ovarian cancer recurrence. Computed tomography shows large cystic-solid pelvic tumor with fluid/gas level and adjacent rectum as indirect sign of fistula.

The median diameter of the relapsed tumor was 89 mm (range 36–130 mm). In all cases, fistulas formed between the tumor and large bowel. In three cases, fistulas formed between the rectum, including two between the sigmoid colon, one sigmoid and descending colon, one between the cecum and ascending colon, and one between the sigmoid colon and left ureter. On CT, in all cases, the fistulas were not directly visible, with only indirect signs of the fistula observed, such as infiltration of the intestine by the tumor, which we observed as vanishing of the fatty tissue between the tumor and the description of the intestinal wall, disruption of the intestinal wall, or the presence of gas in the tumor. In five patients, we observed thickening of the intestinal wall associated with a fistula (Figure 1, Figure 2 and Figure 3).

To confirm the suspected fistula, the patients were referred for MRI. In all cases, the fistula was visible on the MR images, showing hypointensity on the T2 and T1 post-contrast sequences but no restricted diffusion on the DWI sequence. The fistula in one of the patients was hyperintense on T2 images and hypointense on T1-weighted images and showed restricted diffusion on DWI.

**Figure 4:**
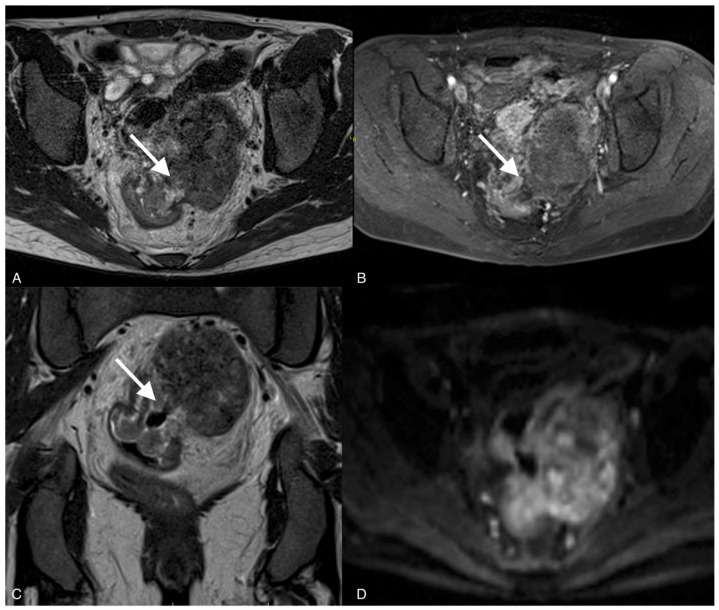
Images of a 37-year-old woman with a recurrent tumor of high-grade serous ovarian cancer with fistula between tumor and sigmoid colon. MRI T2-weighted images obtained in axial plane (**A**), coronal plane (**B**), and T1 post-contrast (**C**) show a large solid/cystic tumor and the fistula (arrows) between tumor and sigmoid colon. DWI (b1200) shows restricted diffusion in the tumor but not in the fistula (**D**).

**Figure 5:**
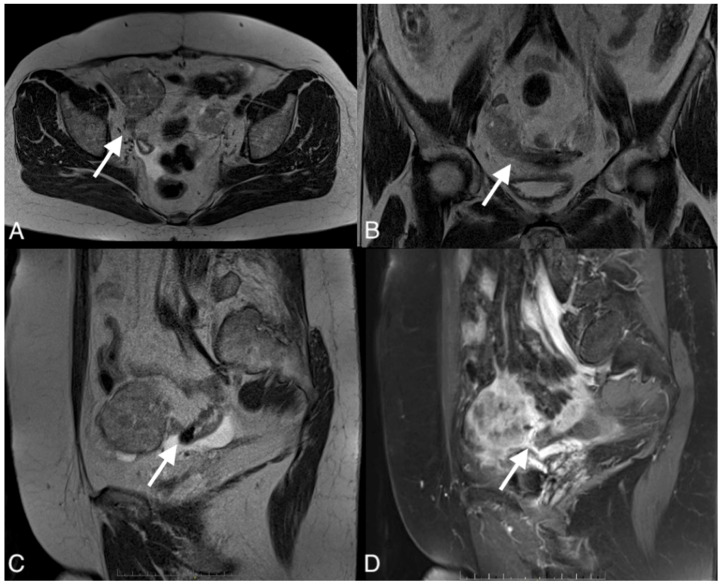
Images of a 67-year-old woman with a recurrent high grade serous ovarian cancer. MRI T1-weighted post-contrast image obtained in the axial plane (**A**), coronal plane (**B**), sagital plane (**C**), and T1-weighted image post-contrast (**D**) show large pelvic tumor and fistula (arrows) between the tumor and sigmoid colon.

Interobserver agreement was 75% for CT and 87.5% for MRI, corresponding to a high level of agreement (Figure 4 and Figure 5).

### 3.2. Surgical Findings

Patients who were qualified for the study underwent secondary cytoreductive surgery. In all eight cases, the fistulas between the tumor and surrounding organs were confirmed as a concomitant sign of cancer relapse. No residual macroscopic tumor remained in seven cases (resection R0-87.5%). In one patient, 1–2 mm implants were left in the mesentery of the ileum (resection R1). During surgery, seven intestinal anastomoses, one colostomy in the descending colon, and one case of tumor resection with fragments of bladder and ureter with implantation of the ureter to the bladder were performed. The types of fistulas, their locations, and the type of surgery are described in Table 4.

Right hemicolectomy was performed over a linear cutter stapler (GIA), anterior rectal resection with a linear stapler, and anastomosis with a trans-rectal circular end-to-end anastomosis (EEA) device. Overall morbidity was 25% (two of eight patients), including fever greater than 38 degrees for three days in two patients and suppuration of the wound in one patient (this patient also had fever over 38 degrees). No bowel-specific morbidity was noted, such as an anastomotic leak, ileus greater than one week, and pelvic abscess.

### 3.3. Oncological Outcomes

Six of our patients experienced another relapse. Two patients who received olaparib as a maintenance treatment were observed for 36 and 28 months after the end of second-line chemotherapy, with no cancer recurrence. One of the patients died. The progression-free survival (PFS) after the end of second-line chemotherapy was 8.4–22.6 months (median 13.4 months). In the group with cytoreduction R0, the median PFS was 15.5 months (12–22 months). The mean survival time after receiving second-line chemotherapy was 56.2 months.

## 4. Discussion

The present study on eight patients showed the imaging and treatment data of a tumor/bowel fistula as a rare form of relapsed ovarian cancer. Patients were qualified for surgery using the AGO score criteria, with R0 resection in seven of eight cases. The median PFS was 13 months, and the mean survival time after receiving second-line chemotherapy was 56 months.

According to the literature, a fistula between the intestine and the tumor is a rare sign of relapsed ovarian cancer and is very difficult to diagnose, as it manifests with nonspecific symptoms. A fistula between a tumor and the intestine can be diagnosed only with the use of imaging methods such as CT and MRI. According to our study, the primary method of diagnosing and confirming a suspected pelvic fistula is MRI, which has also been demonstrated in other studies. MRI allows not only recognition of a fistula between the vagina, bladder, and rectum but also assessment of regional anatomy and relationships with other organs [16,17]. Additionally, gynecological societies recommend MR for patients for whom CT is contraindicated or when findings are inconclusive [18]. Other methods such as CT are also useful, which is usually the first method used to evaluate progression or regression of the disease and causes of the acute abdomen; however, it has limitations when diagnosing the fistula [16].

Fluid-containing fistulas appear as a high signal on T2-weighted images and STIR, while gas-containing fistulas have a low signal in all sequences and demonstrate contrast enhancement. Old, healed fistulas typically demonstrate low T1 and T2 signals without contrast enhancement, reflecting fibrosis [19]. Our study showed that fistulas were hypointense on T2 sequences and hypointense on T1 sequences, and the best sequences to diagnose fistulas on MRI were T2 sequences and late arterial phase post-contrast T1 sequences, which suggest no active inflammatory processes or necrosis.

The study showed that the DWI sequence was not superior to the other sequences in diagnosing fistulas on MRI, except for one of the patients in whom an acute inflammatory process was involved. Recent studies have shown that diffusion-weighted images are useful for diagnosing fistulas in the pelvis and determining whether they involve acute inflammation. However, DWI alone was not superior to T2 and T1 sequences with contrast [20]. DWI can show restricted diffusion in the tumor, which has an important role in the detection, characterization, and definition of local tumor spread [17].

On computed tomography, we observed only indirect imaging signs of fistulas, such as infiltration of the intestine by the tumor, which we observed as vanishing of the fatty tissue between the tumor and the intestine, disruption of the intestinal wall, and gas in the tumor. Computed tomography’s limitation is evident when evaluating local tumor spread due to its lower soft tissue resolution. In other studies, CT was very useful for diagnosis, revealing indirect signs of fistulas, such as obvious thickening of the tumor wall and an air-fluid level within the tumor, suggesting that the ovarian tumor might have communication with the digestive tract; however, they also did not observe fistulas directly [11].

In the literature, only a few case studies have described fistulation associated with ovarian cancer.

The observed patients were older than 50 years and showed symptoms similar to those in our study: abdominal pain and bloody diarrhea [11]. The organs involved in the fistula were the bladder, small intestine, and large intestine; however, in our study, we observed patients with a fistula between the tumor and large intestines [21].

Proposed mechanisms of fistula formation include torsion of the tumor with resultant ischemia and pressure necrosis, infection with adhesion to and eventual rupture into an adjacent organ, and malignant transformation with direct invasion [22].

The lack of bowel-specific morbidity in our group confirms the thesis that such operations can be safely performed by teams of experienced gynecologic oncologists. This finding is confirmed by other studies comparing operations performed by gynecologic oncologists and intestinal surgeons. However, the data are only for primary cytoreduction [23,24].

In our study, the mean PFS for all patients was 13,4 months, but for the subpopulation with complete secondary cytoreduction surgery (R0), it was 15.5 months. Despite a high percentage of complete cytoreduction 87.5, the PFS in our study was shorter than that in the DESKTOP I-III study (18.4 months), the CALIPSO study (18.2 months), the GOG 213 study (18.9 months), and the SOC-1 study (17.4 months). In the last two trials, the median PFS rates for patients with complete secondary cytoreduction resection (R0) were 22.4 months and 19.2 months, respectively [25,26]. Therefore, we can hypothesize that ovarian cancer relapse associated with a fistula between the bowel and tumor accounts for the poor prognosis. Of course, this is only a hypothesis.

The limitation of the present study is the sample size, which was small because a rare form of ovarian cancer recurrence was examined. Furthermore, this was a single-center study.

## 5. Conclusions

In patients with suspected EOC recurrence with clinically suspected fistula, CT scan is not sufficient. In CT, the presence of a fistula is suspected, based on indirect symptoms. MRI, as a method with much greater tissue resolution, confirms the diagnosis. In addition, MRI can identify the point of the tumor/bowel junction. This is especially true with a large infiltration, covering several intestinal parts.

Bowel resection with a simultaneous anastomosis is a good and safe solution for these patients. However, appropriate qualification for the procedure is necessary, allowing for surgery without residual macroscopic disease (R0 surgery).

Due to the small number of cases, our results cannot be generalized. We treat them as hypothetical and encourage other researchers to verify them in a larger study.

## Figures and Tables

**Table 1:** Patient characteristics.

Clinical Data	Number (Median)
Number of patients	8
Age	37–78 (59)
FIGO stage	
II	1
III	6
IV	1
Histopathology of EOC	
high-grade serous	5
low-grade serous	1
carcinosarcoma	1
clear cell carcinoma	1
Primary surgical treatment	
optimal debulking surgery	4
suboptimal surgery	4
Adjuvant therapy	
paclitaxel + carboplatin	8
bevacizumab *	4
Treatment response (after first-line chemotherapy)	
CR	8
PR	0
SD	0
PD	0
PFS (months)	6–27 (17)
Diagnosis of recurrence/clinical symptoms	
CT/MRI	8
bowel obstruction	1
rectal bleeding/bloody diarrhea	2
abdominal pain	3
weight loss	3

EOC—epithelial ovarian cancer; CR—complete remission; PR—partial remission; SD—stable disease; PD—progression disease, PFS—progression-free survival; CT—computed tomography; MRI—magnetic resonance imaging. * According to the results of the ICON study, patients at high risk of relapse received maintenance treatment with bevacizumab at a dose of 7.5 mg/kg every three weeks for a total of 18 courses or until progression [5].

**Table 2:** Technical parameters of computed tomography.

Current	120 kV
Voltage	250 mA
Slice Thickness	1.25–2.5 mm
FOV	33.7 × 39.9 cm
Rotation time	0.7 s
Pitch	0.984:1

**Table 3:** Some of the parameters of the 1.5T Magnetic Resonance (Magnetom Avanto, Siemiens AG, Erlangen) used in the study.

Parameter	T2 TSE	T2 Tirm	VIBE T1 GRE 3D	T2 TSE Fat-Sat	DWI EPI b = 5,050,010,001,500 mm^2^/s	T1 TSE Fat-Sat	T2 TSE BLADE (Fat-Sat) SPAIR	T1 GRE (In- and Out-Phase)
FOV [mm]	360	360	360	360	360	360	360	360
Orientation	axial, sagittal, coronal	axial	axial	axial	Axial	axial, coronal, sagittal	axial, coronal	axial
Repetition time [ms]	3190	6100	3.05	4250	4240	666	2300	125
Echo time [ms]	116	39	1.13	114	73	10	116	1:2.22 2:4.92
Flip angle [deg.]	137	150	10	137	90	90	150	70
Breath hold	no	no	no	no	No	no	yes	no
Matrix	256 × 236	320 × 320	188 × 216	256 × 256	128 × 84	256 × 168	256 × 256	512 × 384
Slice thickness [mm]	3	4	3	3	6	3	4	6
Number of signal averages	1	1	1	1	4	1	1	1

TSE—turbo spin-echo; TIRM—turbo inversion recovery magnitude; VIBE—volumetric interpolated breath-hold examination; GRE—gradient echo sequence; EPI—echo planar imaging; DWI—diffusion-weighted imaging; BLADE—in MRI system for Siemens name for periodically rotated overlapping parallel lines with enhanced reconstruction (PROPELLER).

**Table 4:** The types and localization of the fistulas and the type and outcome of the surgical procedure.

Patient’s Number	Localization	Surgical Procedure	Anastomosis	Surgical Outcome
1	sigmoid	anterior resection	end to end	R-0
2	sigmoid and descending colon	left hemicolectomy	end to end	R-0
3	rectum	anterior resection	end to end	R-0
4	rectum	Hartman’s operation	colostomy	R-1 (1–2 mm peritoneal implants in the mesentery)
5	cecum and ascending colon	right hemicolectomy	side to side	R-0
6	sigmoid and left ureter	anterior resection and resection of part of the ureter with implantation into the urinary bladder	end to end	R-0
7	rectum	anterior resection	end to end	R-0
8	sigmoid	anterior resection	end to end	R-0

## Data Availability

The data presented in this study are available upon request from the corresponding authors.
